# Debridement or Microfracture for Full-Thickness Cartilage Lesions in Anterior Cruciate Ligament Reconstruction: A 10-Year Cohort Study of 326 Patients in Norway and Sweden

**DOI:** 10.1177/23259671251381340

**Published:** 2025-10-28

**Authors:** Stian Kjennvold, Jan Harald Røtterud, Magnus Forssblad, Lars Engebretsen, Asbjørn Årøen, Svend Ulstein

**Affiliations:** † Akershus University Hospital, Lørenskog, Norway; ‡ Institute for Clinical Medicine, University of Oslo, Oslo, Norway; § Oslo Sports Trauma Research Center, Oslo, Norway; ‖ Oslo University Hospital, Oslo, Norway; ¶ Stockholm Sports Trauma Research Center, Karolinska Institute, Stockholm, Sweden; # Norwegian Knee Ligament Registry, Bergen, Norway; Investigation performed at the Department of Orthopedic Surgery, Akershus University Hospital, Lørenskog, Norway

**Keywords:** anterior cruciate ligament, knee, articular cartilage, debridement, microfracture

## Abstract

**Background:**

It is well-established that patients undergoing anterior cruciate ligament reconstruction (ACLR) have a less favorable prognosis if a concomitant full-thickness cartilage lesion is present at the time of surgery. However, the long-term benefits of surgically addressing these cartilage lesions remain uncertain.

**Purpose:**

To evaluate whether the most used surgical treatments for cartilage lesions influenced patient-reported outcomes 10 years after ACLR.

**Study Design:**

Cohort study; Level of evidence, 2.

**Methods:**

This study was based on a cohort of all patients with primary unilateral ACLR in the Norwegian and Swedish knee ligament registries from January 2005 through December 2008 (n = 15,783). A total of 1012 (6.4%) patients had ≥1 full-thickness cartilage lesion (International Cartilage Regeneration & Joint Preservation Society grade 3 or 4) at the time of reconstruction. Of these, 644 patients met the inclusion criteria. At a mean (± SD) follow-up of 10.1 ± 0.2 years, 326 (51%) of the patients had completed the Knee injury and Osteoarthritis Outcome Score (KOOS). The treatment categories documented in the Scandinavian knee ligament registries were microfracture, debridement, no treatment, or other/unknown. Patients who did not receive surgical treatment for their full-thickness cartilage lesions served as the reference group and were compared with those who underwent microfracture or debridement. Multiple linear regression, with adjustment for possible confounders, was used to evaluate associations between surgical treatment of cartilage lesions and patient-reported outcomes for all KOOS subscales 10 years after ACLR.

**Results:**

Of the 326 patients available at the 10-year follow-up, 182 (56%) had not received surgical treatment for their cartilage lesions. A total of 68 (21%) patients had been treated with debridement, and 76 (23%) patients had been treated with microfracture. No significant association with KOOS scores was observed for debridement or microfracture compared with the reference group without surgical treatment of their cartilage lesion. The unadjusted data showed a trend toward poorer outcomes with microfracture compared with debridement, but this difference was not statistically significant.

**Conclusion:**

Debridement and microfracture showed no significant association with patient-reported outcomes 10 years after ACLR in patients with concomitant full-thickness cartilage lesions, compared with those who did not receive surgical treatment for their cartilage lesion.

Focal cartilage lesions are common in anterior cruciate ligament (ACL)–injured knees with a reported prevalence of around 25% of patients undergoing ACL reconstruction (ACLR) in national registries.^15^ Cartilage lesions can be preexisting, be caused by shear forces during the initial trauma, or develop due to recurrent instability.^8^ There is a growing body of evidence that concomitant cartilage injuries could exacerbate symptoms and have a negative effect on the patient-reported outcomes (PROs) after ACLR.^4,6,23,26^ The presence of full-thickness cartilage lesions, extending down to or into subchondral bone, has also been shown to increase the risk of early onset osteoarthritis after ACLR.^2,28^ Addressing full-thickness injuries surgically at the time of ACLR could therefore be warranted.

The past decades have seen several treatment options emerge, ranging from simple debridement to advanced cell-based treatments. However, the optimal treatment for focal cartilage lesions remains unknown. Furthermore, there is a lack of evidence on the long-term effect of these different treatment modalities and their role in ACLR. Although not all cartilage lesions are treated surgically, microfracture (MF) and debridement are the most frequently used treatment modalities for focal cartilage lesions in the setting of ACLR because of their relative simplicity, arthroscopic accessibility, and cost-effectiveness. MF involves perforating subchondral bone with the aim of releasing bone marrow cells and growth factors to aid in formation of fibrocartilage in the defect. Debridement, on the other hand, involves removal of loose fragments and stabilization of the lesion edges and is traditionally considered a palliative rather than reparative or regenerative procedure.

While only a small number of studies have compared MF and debridement in the setting of ACLR, evidence to support the use of these treatment modalities is lacking and knowledge about the PROs is limited to short- and midterm results.^10,24,25^ In addition, Røtterud et al^24^ demonstrated a significantly negative effect of microfracture on the Knee injury and Osteoarthritis Outcome Score (KOOS) subscales Sport and Recreation and knee-related Quality of Life compared with leaving the cartilage lesion untreated 2 years after ACLR.

The primary objective of this nationwide registry-based cohort study was to assess the long-term patient-reported effect of surgical debridement or MF as compared with nonoperative treatment of focal cartilage lesions at the time of ACLR.

## Methods

The current study is a binational, prospective cohort study including patients from the Norwegian National Knee Ligament Registry (NKLR) and the Swedish National Knee Ligament Register (SKLR). The registries were established in 2004 (Norway) and 2005 (Sweden) and designed to collect information prospectively on all cases of knee ligament reconstruction surgery nationwide.^9^ The Swedish registry was based on the Norwegian to facilitate collaboration, and there are no major between-country differences with regard to demographics or treatment strategies.^15^ The registration in both countries is voluntary, but baseline registry participation for primary ACLR has been reported to exceed 90%, reflecting close to complete national coverage at the time of surgery.^9,14^

Data in the registries are collected both from the attending surgeon and the patients. Patients fill out a translated and validated version of the KOOS preoperatively at index surgery and then again after 2, 5, and 10 years. The surgeon enters surgery-specific variables, including details on any concomitant ligament, meniscal, or cartilage lesions. Cartilage lesions are described using the International Cartilage Regeneration & Joint Preservation Society (ICRS) classification system.^3^ The system contains information about lesion characteristics such as area, depth, and localization. Area of the lesion is dichotomized to either <2 cm^2^ or ≥2 cm^2^, whereas lesion depth is graded from 1 to 4. Grades 1 and 2 are used for lesions ranging from superficial down to <50% of cartilage thickness. Grades 3 and 4 are commonly referred to as full-thickness cartilage lesions and contain lesions ranging from >50% of cartilage depth to lesions involving subchondral bone.

KOOS is a PRO measure that contains 5 subscales that all give an aggregated score to assess knee function. The 5 domains are Pain, Symptoms, Activities of Daily Living, Sport and Recreation, and knee-related Quality of Life. Each subscale is scored from 0 to 100, with 100 representing no knee-related problems. The KOOS is commonly used to assess the effect of knee injuries and has been validated for use in patients with knee cartilage lesions as well as ACL injuries.^1,22^

### Study Population

We conducted a 10-year follow-up of all patients who underwent a unilateral primary ACLR and were included in the NKLR or SKLR between January 1, 2005, and December 31, 2008. During this time frame, a total of 15,783 patients were prospectively registered. For the current study, a full-thickness cartilage lesion was defined as a grade 3 or grade 4 lesion according to the ICRS criteria. Patients with >1 concomitant cartilage lesion were categorized according to the lesion with the highest ICRS grading.

A total of 1012 patients were listed in the NKLR or SKLR with a concomitant full-thickness (ICRS grade 3 or 4) cartilage lesion and were thus potentially eligible for our study. This patient cohort has previously been described in studies that have compared the effect of debridement or microfracture of full-thickness cartilage lesions 2 and 5 years after ACLR.^24,25^

**Figure 1.**
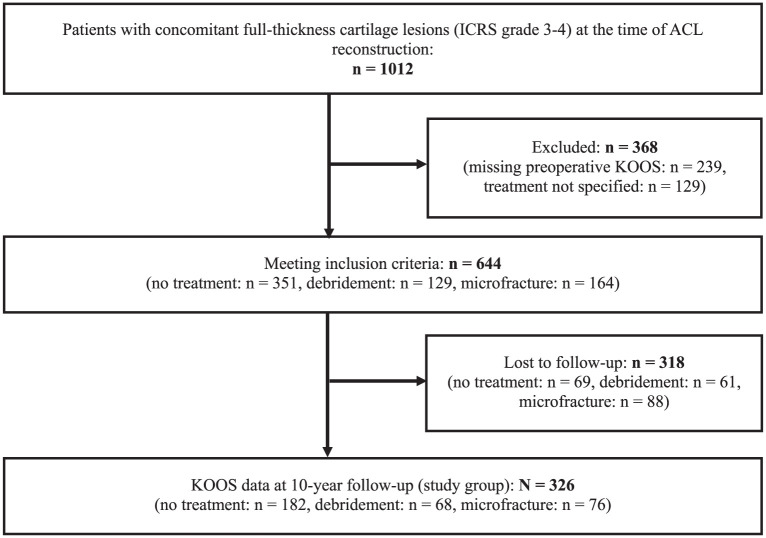
Study inclusion flowchart for comparison of no surgical treatment to microfracture or debridement of focal cartilage lesions at anterior cruciate ligament (ACL) reconstruction. ICRS, International Cartilage Regeneration & Joint Preservation Society; KOOS, Knee injury and Osteoarthritis Outcome Score.

In addition to having a full-thickness cartilage lesion, eligible patients had to have preoperative KOOS values and be registered as undergoing no treatment, debridement, or MF of the cartilage lesion to meet the inclusion criteria. Of the 1012 patients, 368 patients did not meet the inclusion criteria because of missing preoperative KOOS data (n = 239) or because information on cartilage lesion treatment was missing or listed as other than no treatment, debridement, or MF (n = 129). Of the 644 patients who met the inclusion criteria, 318 (49.4%) were lost to follow-up because of missing KOOS values after 10 years. This left 326 patients (50.6%) in the study group, available for analysis at long-term follow-up (Figure 1).

### Baseline Characteristics

The study group and those lost to follow-up were similar for most variables registered in NKLR and SKLR apart from small deviations in age and sex. The group lost to follow-up had a higher proportion of men and a slightly younger age than the study group. Detailed baseline demographics for the study group and those lost to follow-up group are outlined in Table 1.

**Table 1:** Baseline Demographics at the Time of ACL Reconstruction for the Study Group Compared With Patients Lost to Follow-up*^*a*^*

	Study Group (n = 326)	Lost to Follow-up (n = 318)
Age at surgery, y, median (range)	36 (14-64)	32 (14-56)
Time from injury to surgery, mo, median (range)	14 (1-483)	18 (0-360)
Female sex	142 (43.6)	97 (30.5)
Previous ipsilateral knee surgery	127 (39.0)	125 (39.3)
Concomitant ligament injury	33 (10.1)	24 (7.5)
Concomitant meniscal lesion	177 (54.3)	205 (64.5)
Meniscal resection	140 (42.9)	155 (48.7)
ACL graft		
Hamstring tendon	245 (75.2)	252 (79.2)
Bone–patellar tendon–bone	76 (23.3)	59 (18.6)
Other/unknown	5 (1.5)	3 (0.9)
Depth (ICRS grade 4)	85 (26.1)	85 (26.7)
Area of cartilage lesion		
<2 cm^2^	160 (49.1)	138 (43.4)
≥2 cm^2^	161 (49.4)	175 (55.0)
Not reported	5 (1.5)	5 (1.6)
Location of cartilage lesion		
Patella	15 (4.6)	2 (0.6)
Trochlea	22 (6.7)	11 (3.5)
Medial femoral condyle	227 (69.6)	227 (71.4)
Lateral femoral condyle	37 (11.3)	39 (12.3)
Medial tibial plateau	8 (2.5)	8 (2.5)
Lateral tibial plateau	17 (5.2)	12 (3.8)
Preoperative KOOS score		
Pain	69.7 ± 19.2	70.0 ± 19.4
Symptoms	67.7 ± 19.2	66.8 ± 18.5
Activities of Daily Living	77.7 ± 19.9	78.5 ± 18.9
Sport and Recreation	35.7 ± 26.7	36.4 ± 28.3
Knee-Related Quality of Life	30.5 ± 18.2	30.9 ± 18.5

a Data are presented as mean ± SD or n (%) unless otherwise indicated. ACL, anterior cruciate ligament; ICRS, International Cartilage Regeneration & Joint Preservation Society; KOOS, Knee injury and Osteoarthritis Outcome Score.

When stratified by the 3 treatment options—no surgical treatment, debridement, and MF—the groups also demonstrated baseline equivalence for most variables (Table 2). However, there was a higher proportion of grade 4 cartilage lesions in the MF group, compared with the no treatment and debridement group. There was also a tendency for MF to be performed on smaller area lesions than debridement or no surgical treatment. The majority of cartilage lesions were found on the medial femoral condyle.

**Table 2:** Baseline Characteristics of Study Group Stratified by Treatment Modality of the Focal Cartilage Lesion*^*a*^*

	No Treatment (n = 182)	Debridement (n = 68)	Microfracture (n = 76)
Age at surgery, y, median (range)	37 (14-59)	36 (15-64)	35 (15-62)
Female sex	82 (45.1)	26 (38.2)	34 (44.7)
Time from injury to surgery, mo, median (range)	15 (1-322)	12 (1-242)	14 (1-483)
Previous ipsilateral knee surgery	83 (45.6)	21 (30.9)	23 (30.3)
Concomitant ligament injury	19 (10.4)	8 (11.8)	6 (7.9)
Concomitant meniscal lesion	100 (55.0)	34 (50.0)	43 (56.6)
Meniscal resection	77 (77.0)	31 (91.2)	32 (74.4)
ACL graft			
Hamstring tendon	135 (74.2)	51 (75.0)	59 (77.6)
Bone–patellar tendon–bone	46 (25.3)	13 (19.1)	17 (22.4)
Other/unknown	1 (0.5)	4 (5.9)	0 (0)
Depth of cartilage lesion (ICRS grade 4)	39 (21.4)	8 (11.8)	38 (50.0)
Area of cartilage lesion			
<2 cm^2^	84 (46.2)	31 (45.6)	45 (59.2)
≥2 cm^2^	93 (51.1)	37 (54.4)	31 (40.8)
Not reported	5 (2.7)	0 (0)	0 (0)
Location of cartilage lesion			
Patella	8 (4.4)	6 (8.8)	1 (1.3)
Trochlea	13 (7.1)	2 (2.9)	7 (9.2)
Medial femoral condyle	122 (67.0)	48 (70.6)	57 (75.0)
Lateral femoral condyle	20 (11.0)	10 (14.7)	7 (9.2)
Medial tibial plateau	4 (2.2)	2 (2.9)	2 (2.6)
Lateral tibial plateau	15 (8.2)	0 (0)	2 (2.6)
Preoperative KOOS score			
Pain	69.8 ± 19.4	72.3 ± 19.2	67.0 ± 18.5
Symptoms	68.0 ± 19.5	69.9 ± 17.3	64.9 ± 20.1
Activities of Daily Living	77.9 ± 19.6	80.9 ± 18.0	74.2 ± 22.0
Sport and Recreation	36.6 ± 27.0	39.4 ± 26.3	30.3 ± 26.0
Knee-Related Quality of Life	30.1 ± 18.9	32.2 ± 16.4	30.0 ± 18.3

a Data are presented as mean ± SD or n (%) unless otherwise indicated. ACL, anterior cruciate ligament; ICRS, International Cartilage Regeneration & Joint Preservation Society; KOOS, Knee injury and Osteoarthritis Outcome Score.

### Statistical Analysis

Stata SE Version 18.0 (Stata Corp LLC) was used for all statistical analyses. Differences were considered statistically significant for *P* values <.05. All crude mean KOOS scores and regression coefficient estimates (βs) are presented with 95% CIs.

To evaluate the association between the different treatment modalities for concomitant cartilage lesions and the 10-year PROs as measured by KOOS, we employed multivariable linear regression. Separate analyses were made for each KOOS subscale, and the independent variables included in our regression model were age at surgery, sex, time from injury to surgery, previous ipsilateral knee surgery (yes/no), concomitant ligament or meniscal injury (yes/no), type of ACL graft (bone–patellar tendon–bone/hamstring/other), area (<2 cm^2^ or ≥2 cm^2^), lesion depth (ICRS grade 3 or 4) and preoperative KOOS. The patients not receiving any surgical treatment for their concomitant cartilage lesion at the time of ACLR were used as a control group when comparing the KOOS outcome for patients treated with MF or debridement.

Although both ICRS grade 3 and 4 lesions were classified as full thickness, separate analyses were made for each of the 2 grades to further evaluate whether lesion depth influenced the effectiveness of surgical intervention on long-term PROs. The results are presented with both adjusted and unadjusted values to indicate the effect of each possible confounding factor.

To establish the proportion of patients that would regard their outcome as good, satisfactory, or poor, previously established threshold values for Patient Acceptable Symptom State (PASS) and Treatment Failure (TF) for ACLR patients were utilized.^12,21,27^ PASS and TF are descriptive categorizations designed to assess patient-perceived symptom acceptability and treatment success, rather than outcomes for hypothesis testing.^12^ They provide a structured way to interpret the clinical relevance of KOOS subscale scores. PASS and TF proportions are presented here for the KOOS subscales Sport and Recreation and knee-related Quality of Life, because they were regarded as the most relevant outcome measures for ACLR patients.^20^ Patients were categorized as PASS if they reached or exceeded a predefined threshold value of 72 for Sport and Recreation and 73 for Quality of Life. TF was defined as a score of ≤28 points for both subscales. The patients between these thresholds were classified as “neither PASS nor TF.”

## Results

**Figure 2.**
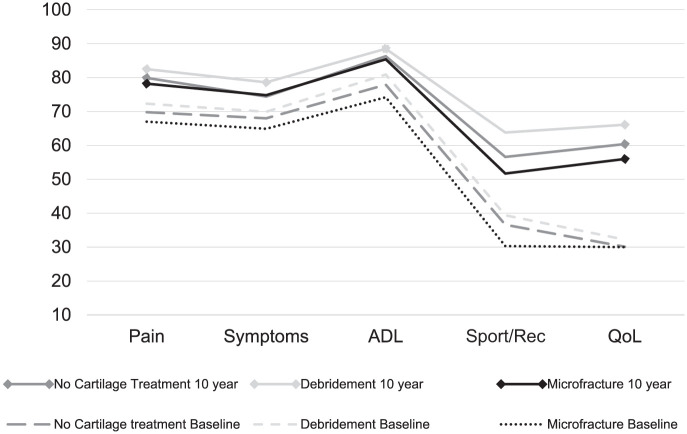
Profiles of mean unadjusted scores of the Knee injury and Osteoarthritis Outcome for the patients without surgical treatment, debridement, or microfracture of full-thickness cartilage lesion at the time of anterior cruciate ligament reconstruction. Baseline scores compared with values from the 10-year follow-up. ADL, Activities of Daily Living; QoL, knee-related Quality of Life; Sport/Rec, Sport and Recreation.

At a mean (± SD) 10.1 ± 0.2-year follow-up, 326 (50.6%) of the patients completed KOOS. The mean (± SD) age at follow-up was 36.0 ± 10.7 years. At the time of ACLR, 182 (55.8%) patients received no surgical treatment of their cartilage lesion, 68 (20.9%) were treated with debridement, and 76 (23.3%) were treated with MF. All 3 treatment groups showed improvement in KOOS-reported outcomes from preinjury level to the 10-year follow-up. The change in KOOS for all subscales from baseline to the 10-year follow-up is outlined in Figure 2.

At the 10-year follow-up after ACLR, the patients treated with MF overall reported inferior crude mean values for all KOOS subscales when compared with patients treated with debridement or no surgical treatment of the cartilage lesion. The patients in the debridement group had the highest mean scores for all KOOS subscales (Table 3).

**Table 3:** Crude KOOS for All Subscales Stratified by Treatment of Concomitant Cartilage Lesion at 10-Year Follow-up After ACL Reconstruction^**a**^

KOOS Subscale	No Treatment (n = 182)	Debridement (n = 68)	Microfracture (n = 76)
Pain	79.9 (76.9-82.9)	82.5 (77.9-87.1)	78.2 (73.6-82.4)
Symptoms	74.4 (71.3-77.5)	78.6 (73.5-83.7)	74.8 (70.0-79.6)
ADL	86.3 (83.6-89.0)	88.5 (84.3-93.7)	85.4 (81.1-89.8)
Sport/Rec	56.6 (51.8-61.4)	63.8 (57.1-70.6)	51.7 (44.7-58.7)
QoL	60.4 (56.3-64.4)	66.1 (59.5-72.2)	56.0 (50.0-62.0)

a Data are presented as mean (95% CI). ACL, anterior cruciate ligament; ADL, Activities of Daily Living; KOOS, Knee injury and Osteoarthritis Outcome Score; QoL, knee-related Quality of Life; Sport/Rec, Sport and Recreation.

The regression analyses comparing debridement and MF with the reference group without surgical treatment of cartilage lesions were based on KOOS scores for all subscales at the 10-year follow-up rather than the changes from baseline. These analyses revealed no significant unadjusted or adjusted associations between cartilage treatment and KOOS 10 years after ACLR (Table 4).

**Table 4:** Unadjusted and Adjusted Regression Analyses of the Associations Between All KOOS Subscales and Treatment of Full-Thickness Cartilage Lesions at 10-Year Follow-up After ACL Reconstruction*^*a*^*

	n	Debridement			Microfracture		
KOOS Subscale		β	95% CI	*P*	β	95% CI	*P*
Pain							
Unadjusted	326	2.6	(–3.1 to 8.3)	.37	–1.7	(–7.1 to 3.8)	.55
Adjusted	307	1.9	(–3.5 to 7.4)	.49	0.9	(–4.4 to 6.3)	.73
Symptoms							
Unadjusted	326	4.2	(–1.7 to 10.1)	.16	0.4	(–5.1 to 6.1)	.89
Adjusted	308	4.3	(–1.5 to 10.1)	.14	3.4	(–2.4 to 9.2)	.25
Activities of Daily Living							
Unadjusted	326	2.1	(–3.0 to 7.3)	.41	–0.9	(–5.8 to 4.1)	.73
Adjusted	307	1.7	(–3.2 to 6.5)	.50	1.7	(–3.1 to 6.4)	.49
Sport and Recreation							
Unadjusted	326	7.2	(–1.5 to 15.9)	.11	–4.9	(–13.3 to 3.5)	.25
Adjusted	308	5.8	(–2.8 to 14.4)	.19	–0.8	(–9.4 to 7.9)	.86
Knee-Related Quality of Life							
Unadjusted	325	5.7	(–1.9 to 13.3)	.14	–4.4	(–11.7 to 3.0)	.24
Adjusted	307	4.3	(–3.2 to 11.9)	.26	–3.1	(–10.7 to 4.4)	.41

a Adjusted for age at surgery, sex, time from injury to surgery, previous ipsilateral knee surgery, concomitant ligamentous or meniscal injury, type of ACL graft, area (<2 cm^2^ or ≥2 cm^2^), lesion depth (ICRS grade 3 or 4), and preoperative KOOS. Patients without treatment of their full-thickness cartilage lesion are used as reference. ACL, anterior cruciate ligament; ICRS, International Cartilage Regeneration & Joint Preservation Society; KOOS, Knee injury and Osteoarthritis Outcome Score.

**Figure 3.**
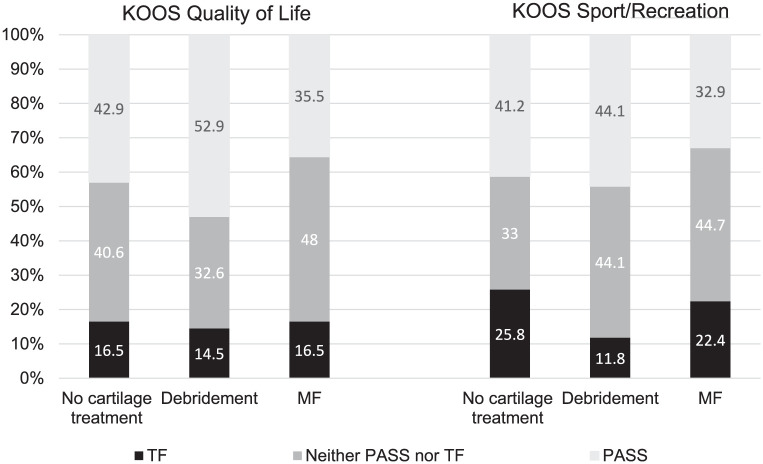
Proportion of patients reaching Patient Acceptable Symptom State (PASS) or scoring at or below threshold for treatment failure (TF) for the Knee injury and Osteoarthritis Outcome Score (KOOS) subscales knee-related Quality of Life and Sport/Recreation when stratified by treatment of cartilage lesion. MF, microfracture.

The proportions of patients achieving PASS or falling below the predetermined TF score are shown in Figure 3. There was a higher proportion of patients reaching PASS for both KOOS Sport and Recreation and knee-related Quality of Life subscales in the group treated with debridement compared with the MF or no surgical treatment groups. The lowest proportion of patients reaching PASS was found in the MF group for both subscales.

## Discussion

The main finding of the present study was that treatment of concomitant cartilage lesions with MF or debridement at the time of ACLR was not significantly associated with 10-year KOOS outcomes compared with patients whose cartilage lesions were left untreated. This suggests that surgical intervention for full-thickness cartilage lesions at the time of ACLR, at least using MF or debridement, may not provide any long-term benefit in terms of PROs.

Although a previous study on this cohort found that microfracture was associated with significantly lower KOOS Quality of Life and Sport and Recreation subscale scores at the 2-year follow-up, the difference between groups seemed to diminish over time.^24,25^ The proportion of patients lost to follow-up was fairly consistent across time points (45% at 2 years, 43% at 5 years, and 49% at 10 years), suggesting that the modest increase in attrition alone is unlikely to account for the diminishing between-group differences. There was a trend toward inferior KOOS scores for MF compared with debridement for the same 2 subscales both at the 5-year follow-up and the current 10-year follow-up however, the difference between groups was not significant. This in contrast to other studies, not limited to ACLR patients, that have reported good short- to midterm outcome of MF for full-thickness cartilage lesions but a deterioration over time.^7,16^

It is, however, important to consider that treatment outcomes for ACL-injured knees may differ from patients with isolated cartilage damage due to the biological response triggered by intra-articular trauma. During ACL injury or reconstruction, there is a release of growth factors and progenitor cells that could potentially influence healing of cartilage lesions similar to what has been shown for meniscal injuries.^17^

This is the largest study cohort to evaluate long-term outcomes after surgical treatment of concomitant cartilage lesions in ACLR. The cohort size and extensive 10-year follow-up ensure robust data that offer new knowledge on how cartilage lesion treatment affects PROs over time. External validity is strengthened using extensive nationwide registries from 2 different countries that have demonstrated a high degree of compliance.^9,14^ Comparison with other knee ligament registries such as the US-based Kaiser Permanente ACLR registry, also support the robustness and generalizability of the current results.^15^

Data in the registries are collected independently of the research question, and selection bias or recall bias is thus limited. The use of a control group further strengthens the comparison of treatment modalities and their effect on KOOS 10 years after ACLR.

### Limitations

The main limitation in this study is the loss to follow-up of close to 50%. Even though the registries over the 4-year time span of our study included almost 16,000 patients, more than half the initial population were nonresponders and hence lost to follow-up. This introduces the possibility of attrition bias, meaning that the final study sample may not fully represent the initial cohort. Baseline demographics, however, demonstrated good equivalence between groups for most variables and the possible confounders were adjusted for in the regression model. In addition, previous responder analyses from the Danish Knee Ligament Reconstruction Registry and Hospital for Special Surgery in New York found that the nonresponders were comparable with the included patients indicating that the data might be valid despite a high loss to follow-up.^18,19^

The current patient cohort had a higher age and increased time from injury to surgery compared with the complete registry population at the time. These differences likely reflect clinical practice during the inclusion period. Although causality cannot be determined, delayed reconstruction and higher age have both been associated with an increased prevalence of intra-articular pathology in previous studies.^8^ In addition, higher age and longer time from injury to surgery have been shown to be negative prognostic factors on outcome after cartilage treatment.^5,13^ To account for these potential confounding factors, our multivariate regression analyses included adjustments for age and time from injury to surgery.

We did not derive new PASS and TF thresholds from our own KOOS distributions. However, the thresholds employed were established using anchor-based methods in large ACL-reconstructed cohorts and validated for use 10 years after ACLR.^11,27^ Their patient-reported assessment of acceptable versus failed outcomes in an equivalent population supports the validity and clinical relevance of our findings despite the absence of cohort-specific distributional analyses.

Although similar, there were some baseline differences between groups, including a higher proportion of grade 4 lesions in the MF group. There was no randomization to the different treatment groups, rather the decision to treat any cartilage lesion was made by the attending surgeon. MFs seem to be used more frequently for the deepest lesions but also more frequently on the smaller lesions (<2 cm^2^). This represents a possible selection bias that could skew the results of our analyses. However, the regression analyses were adjusted for lesion depth as a possible confounder.

The observational study design cannot make firm conclusions on causality and a known limitation in registry research is that the data are limited to those included before the research question was defined. For instance, specific procedural details on cartilage lesion treatment were confined to the predefined registry categories, meaning there was no information on how microfracture or debridement was performed. In addition, KOOS was the only outcome measure analyzed, and although adjusted for any major confounders to isolate the effect of cartilage lesion treatment, the selection of independent variables was based on available data, previous literature, and clinical assumption. Crucial variables such as body mass index, smoking status, activity level, and others that could influence outcome were not available at the time of inclusion for this study cohort and alternative outcome measures such as return to sports, knee joint laxity, and conversion to knee arthroplasty were not recorded. Finally, the lack of imaging assessments to quantify any cartilage healing or degeneration over time also limits our analyses and would be important additions in further studies. Nevertheless, the balanced distribution of patient characteristics within treatment groups and adjustment for key confounders strengthen the validity of our comparisons and support that the observed differences reflect the long-term effect of cartilage lesion treatment.

Our findings should be considered when informing patients about their injury and deciding on any surgical treatment of concomitant cartilage lesions in ACLR. The use of MF or debridement has yet to demonstrate any favorable effect on long-term PROs after ACLR. Considering that MF showed significantly adverse effects on the 2-year follow-up, the use of these treatment modalities in ACLR should be cautioned. More research is needed to optimize management of these combined injuries. Emphasis should be put on developing better treatment options or identifying subgroups that might profit from existing treatment.

## Conclusion

ACL-injured patients with concomitant full-thickness cartilage lesions showed no significant association between treatment with debridement or microfracture and 10-year KOOS outcomes after ligament reconstruction. Considering that MF showed significant adverse effects on KOOS scores at 2-year follow-up in the current cohort, MF should be cautioned in the setting of ACLR.
